# A high-throughput microfluidic nanoimmunoassay for detecting anti–SARS-CoV-2 antibodies in serum or ultralow-volume blood samples

**DOI:** 10.1073/pnas.2025289118

**Published:** 2021-04-16

**Authors:** Zoe Swank, Grégoire Michielin, Hon Ming Yip, Patrick Cohen, Diego O. Andrey, Nicolas Vuilleumier, Laurent Kaiser, Isabella Eckerle, Benjamin Meyer, Sebastian J. Maerkl

**Affiliations:** ^a^Institute of Bioengineering, School of Engineering, École Polytechnique Fédérale de Lausanne, 1015 Lausanne, Switzerland;; ^b^Division of Laboratory Medicine, Department of Diagnostics, Geneva University Hospitals and Geneva University, 1205 Geneva, Switzerland;; ^c^Division of Infectious Diseases, Department of Medicine, Geneva University Hospitals and Faculty of Medicine, 1205 Geneva, Switzerland;; ^d^Laboratory of Virology, Division of Laboratory Medicine, Geneva University Hospitals and Faculty of Medicine, 1205 Geneva, Switzerland;; ^e^Center for Emerging Viral Diseases, Geneva University Hospitals and Faculty of Medicine, University of Geneva, 1205 Geneva, Switzerland;; ^f^Centre for Vaccinology, Department of Pathology and Immunology, University of Geneva, 1205 Geneva, Switzerland

**Keywords:** SARS-CoV-2, nanoimmunoassay, high-throughput serology, microfluidics

## Abstract

Novel technologies are needed to facilitate large-scale detection of severe acute respiratory syndrome coronavirus 2 (SARS-CoV-2) specific antibodies in human blood samples. Such technologies are essential to support seroprevalence studies and vaccine clinical trials, and to monitor quality and duration of immunity. We developed a microfluidic nanoimmunoassay (NIA) for the detection of anti–SARS-CoV-2 IgG antibodies in 1,024 samples per device. The method achieved a specificity of 100% and a sensitivity of 98% based on the analysis of 289 human serum samples. To eliminate the need for venipuncture, we developed low-cost, ultralow-volume whole blood sampling methods based on two commercial devices and repurposed a blood glucose test strip. The glucose test strip permits the collection, shipment, and analysis of 0.6 μL of whole blood easily obtainable from a simple finger prick. The NIA platform achieves high throughput, high sensitivity, and specificity based on the analysis of 289 human serum samples, and negligible reagent consumption. We furthermore demonstrate the possibility to combine NIA with decentralized and simple approaches to blood sample collection. We expect this technology to be applicable to current and future SARS-CoV-2 related serological studies and to protein biomarker analysis in general.

## Significance

COVID-19, caused by SARS-CoV-2, led to an unprecedented global health crisis. As the majority of people infected with SARS-CoV-2 have no or only mild symptoms, many cases aren’t captured by direct testing. However, it is important to establish the true spread of the virus by identifying how many people have been exposed. Detection of anti–SARS-CoV-2–specific antibodies in blood samples can help us understand how the pandemic is evolving over time. We developed a sensitive and specific assay that performs 1,024 measurements in parallel. To enable decentralized blood sample collection, the method can detect antibodies in a small drop of blood obtainable by finger pricking, and the blood can be collected and shipped with a simple, low-cost blood glucose test strip.

The emergence of a new coronavirus at the end of 2019, termed severe acute respiratory syndrome coronavirus 2 (SARS-CoV-2), led to an unprecedented global public health crisis (1). Over a year later, it is estimated that SARS-CoV-2 infected over 100 million people worldwide, and 2 million people died of COVID-19, caused by SARS-CoV-2 (2). As SARS-CoV-2 causes mainly mild disease or infection presents without symptoms, many cases are not captured by direct testing in the acute phase of disease (3). However, to estimate infection fatality rate and guide public health decisions, it is of utmost importance to establish the true spread or prevalence of the virus by identifying how many people have been exposed (4, 5).

Detection of anti–SARS-CoV-2 antibodies using highly sensitive and specific assays can help answer these questions. Several seroprevalence studies have already been conducted, demonstrating rather low seroprevalence rates even in areas that were severely affected (6–9). Such data indicate that herd immunity through natural infection is far from being reached. However, these studies are merely snapshots of an evolving situation, both in time and space. Therefore, there is a sustained need for seroprevalence studies to be continuously conducted in order to monitor virus spread and to keep policy makers informed. In addition, tens to hundreds of thousands of blood samples will need to be tested for each SARS-CoV-2 vaccine phase 3 clinical trial, and large numbers of samples will need to be tested to monitor immune responses after a vaccine has been approved and rolled out. Such studies are cumbersome and expensive to perform, as they require large numbers of serum samples obtained by venipuncture and analyzed by highly sensitive and specific immunological assays to classify samples as seropositive or seronegative.

Several assays, such as enzyme-linked immunosorbent assays (ELISA) or chemiluminescent immunoassays (CLIA), are commercially available, but mainly rely on serum drawn by venipuncture. These tests are also rather expensive, with reagent costs on the order of 3 USD to 10 USD per test. Alternatively, in-house ELISAs are difficult to standardize and require high amounts of recombinant antigen, usually around 100 ng per sample (5). Other recently developed methods such as miniaturized high-throughput ELISAs that use low microliter volumes suffer from lower sensitivity (10), and ultrasensitive assays based on digital ELISA have low sample throughput of 68 tests per hour (11). The comparatively high cost of these assays and the reliance on serum samples taken by venipuncture are considerable hurdles to performing large-scale studies under normal circumstances, but especially so during a pandemic, when sample collection can put clinical staff and study participants at risk. Lateral flow assays (LFAs) can be performed at the point of care or at home, requiring only a “drop” of whole blood, but the sensitivity and specificity of these assays is often low (12–14), and LFAs are relatively expensive, at ∼22 USD per test. Furthermore, LFAs provide test results, but no blood samples are being collected which could be used for follow-up analyses.

There is therefore a need for new technologies to supersede existing methods such as ELISA, CLIA, and LFAs. Novel technologies should be capable of high throughput, low reagent consumption, and low cost per test; achieve high sensitivity and specificity; and be compatible with ultralow-volume whole blood samples in the low or even submicroliter range that can be obtained via a simple finger prick. Biomarker detection using dried whole blood on filter paper or other devices would have tremendous advantages, as the sample can be collected by untrained individuals at home. The samples could then be conveniently shipped by regular mail at ambient temperature to a central laboratory for analysis, and test results could be returned electronically via a mobile app or email.

In this study, we developed and validated a nanoimmunoassay (NIA) that analyzes 1,024 samples in parallel on a single microfluidic device the size of a USB stick. NIA reagent consumption and corresponding costs are roughly 1,000 times lower than a standard ELISA. NIA achieved a specificity of 100% and a sensitivity of 98%, based on the analysis of 134 prepandemic negative sera and 155 positive sera from RT-PCR–confirmed positive individuals. NIA performed well for samples obtained more than 20 d post onset of symptoms, and performed equally well for samples obtained less than 20 d past onset of symptoms. We go on to demonstrate that NIA can be used to detect anti–SARS-CoV-2 antibodies in ultralow-volume dried whole blood samples, eliminating the need for venipuncture blood collection. We tested two commercial blood collection devices: Neoteryx’s Mitra^®^ and DBS System SA’s HemaXis^TM^ DB10, and show that it is possible to repurpose low-cost and widely available blood glucose test strips for sample collection and shipment. Samples could be stored up to 6 d at room temperature with minimal sample degradation. All three methods combined with NIA identified more positive samples than a standard ELISA performed on serum samples collected from the same individuals.

## Results

### NIA Development.

**Fig. 1.**
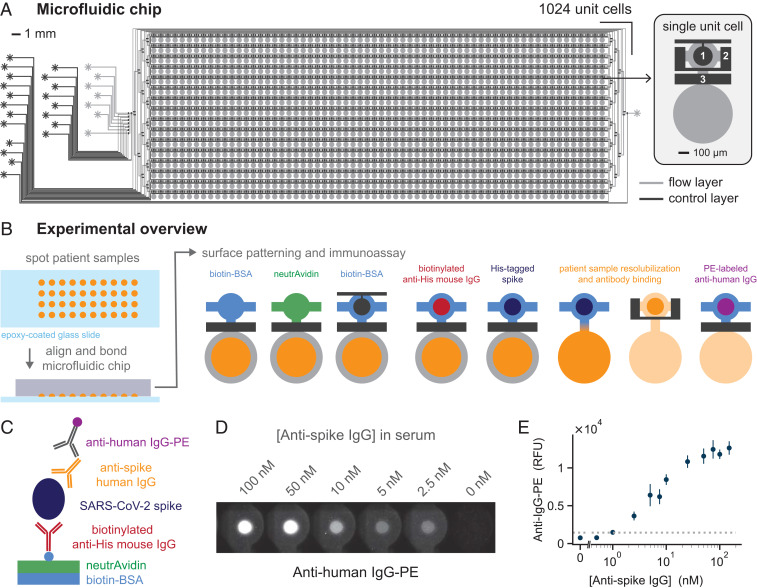
High-throughput microfluidic NIA for anti–SARS-CoV-2 antibody detection. (*A*) The two-layer microfluidic chip design consists of 1,024 unit cells. Each unit cell, in turn, contains two sections: an immunoassay chamber (top) and a spotting chamber (bottom). Control valves include the button (1), sandwich (2), and neck (3) valves. (*B*) A schematic of the experimental process, starting with the spotting of patient samples, followed by chip alignment and bonding, biotin-BSA and neutrAvidin surface patterning, and, lastly, the immunoassay for detection of anti–SARS-CoV-2 antibodies. Surface patterning and immunoassay are shown for a single unit. During the experiment, all unit cells are processed in parallel. (*C*) Schematic of the on-chip sandwich immunoassay. (*D*) Fluorescence images of anti-human IgG-PE signal for a given concentration of anti-spike antibodies present in human serum. (*E*) Quantification of the anti-human IgG-PE signal for a range of anti-spike concentrations spotted. The dashed horizontal line indicates the LOD.

We adapted a mechanically-induced trapping of molecular interactions (MITOMI)-based (15, 16) 1,024-cell NIA device previously applied to vaccine adjuvant screening (17) and the detection of inflammatory and cancer-related protein biomarkers in serum (18) to the detection of anti–SARS-CoV-2 IgG antibodies. The NIA device described here is a simplified version of the original 1,024-cell serum analyzer chip and more similar to the original MITOMI device, with slightly enlarged chambers to accommodate spotted patient samples. The microfluidic device is a standard two-layer polydimethylsiloxane (PDMS) device (19), consisting of a flow and a control layer. Fluids in the flow layer can be manipulated with pneumatic valves formed by the control layer. The device contains 1,024 unit cells, each consisting of an assay and a spotting chamber (Fig. 1*A*). Patient samples are spotted onto an epoxy-coated glass slide using a contact-printing microarray robot, on top of which the PDMS device is aligned and bonded. Patient samples are initially isolated by actuating the neck valve, and the surface of the assay chamber is patterned with biotinylated bovine serum albumin (BSA-biotin) and neutrAvidin, leaving a circular surface region coated with neutrAvidin beneath the MITOMI button. Afterward, biotinylated anti-His antibody is flowed through the device, enabling the subsequent immobilization of a His-tagged SARS-CoV-2 antigen. Patient samples are then solubilized, and the sandwich valves are closed, allowing any SARS-CoV-2–specific antibodies to diffuse into the assay chamber and bind the surface immobilized antigen. The MITOMI button is closed following an incubation period, and any unbound material is washed away. A secondary antibody labeled with phycoerythrin (PE) is flowed to detect the presence of antibodies bound to the SARS-CoV-2 antigen (Fig. 1 *B* and *C*).

We optimized the NIA for detection of anti-spike IgG antibodies in human serum by first testing several different spike antigen variants, and identified the in-house–produced trimerized full-length spike protein as the most suitable antigen (*SI Appendix*, Fig. S1). The use of trimerized full-length spike and spike receptor binding domain both resulted in high signal intensities and low background. We decided on using the trimerized full-length spike protein, as it most closely resembles the natural conformation of the SARS-CoV-2 spike protein. As a technical validation, we added chimeric anti-spike antibody at different concentrations into human serum, spotted each dilution, and showed that NIA could quantify anti-spike antibody concentrations (Fig. 1 *D* and *E*). Based on this sandwich immunoassay, we estimate that our limit of detection (LOD) is around 1 nM IgG. To permit the analysis of patient sera in a standard, biosafety level 1 (BSL-1) laboratory, we developed a simple treatment protocol that renders the sera BSL-1 compatible. This was achieved by conducting a short heat inactivation step, followed by the addition of Triton X-100 to a final concentration of 1% (20). Dried whole blood samples could be safely handled in a BSL-1 environment with the same or similar pretreatment protocol.

### NIA Validation.

**Fig. 2.**
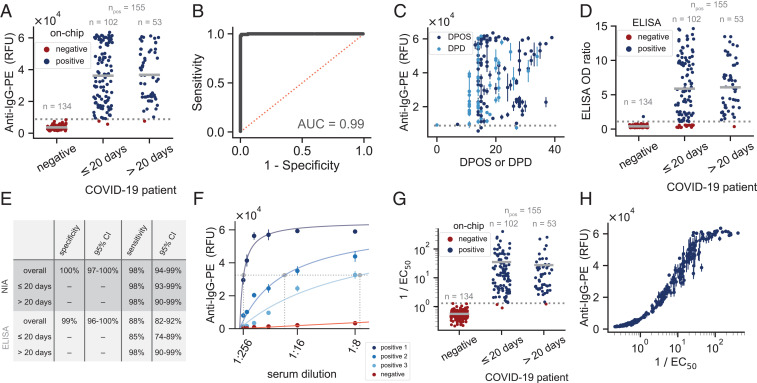
NIA validation. (*A*) NIA anti–IgG-PE signal for serum samples obtained from SARS-CoV-2–negative individuals (obtained in 2013/2014 and 2018) or SARS-CoV-2 RT-PCR–confirmed positive patients. Measurements shown are for a 1:8 dilution of each serum sample. Data points represent mean values of three replicates from a single chip. A total of six devices were run to collect this data. The dashed gray line shows the threshold used for specificity and sensitivity calculations. (*B*) The solid black line represents the ROC curve corresponding to the measurements shown in *A*. The dashed red line corresponds to a classifier with no predictive power with an AUC = 0.5. (*C*) NIA measurements plotted versus days post onset of symptoms or days post diagnosis. Data points represent means ± SD (n=3). The dashed gray line is the same threshold as shown in *A*. (*D*) The same samples were analyzed with a commercial ELISA and the optical density (OD) results plotted as in *A*. The dashed gray line shows the threshold used for specificity and sensitivity calculations. (*E*) Specificity and sensitivity calculations for NIA and ELISA. The dashed gray lines in *A* and *D* indicate the threshold used for the specificity and sensitivity calculations. All calculations are based on classifying samples into the same condition-positive (RT-PCR positive) and condition-negative (prepandemic) groups. (*F*) Examples of curve fits for three positive and one negative patient sample dilution series. The horizontal dashed line indicates half maximal binding, and the three dashed vertical lines correspond to the $EC_{50}$ values determined for each of the positive samples. (*G*) All $1/EC_{50}$ results categorized according to whether the sample was from a negative (prepandemic) or from an RT-PCR–confirmed positive individual. (*H*) Anti–IgG-PE signal from a 1:8 dilution of each serum sample versus its $1/EC_{50}$ value.

We validated the high-throughput NIA for the detection of SARS-CoV-2 anti-spike IgG antibodies with 289 serum samples, collected from 155 PCR-confirmed SARS-CoV-2–infected individuals and 134 prepandemic negative samples collected in 2013/2014 and 2018. Different serum sample dilutions, ranging from no dilution to a 1:256 dilution, were spotted to determine the optimal value (*SI Appendix*, Fig. S2). NIA achieved a maximum specificity and sensitivity of 100% and 98%, respectively, at a serum dilution of 1:8 (Fig. 2*A* and *SI Appendix*, Fig. S2) and a receiver operator characteristic (ROC) curve with an area under the curve (AUC) equal to 0.99 (Fig. 2*B*). As the serum samples were obtained from patients at different days post onset of symptoms (DPOS), we plotted the NIA results according to when samples were obtained (Fig. 2*C*). If DPOS was not known for a given patient, then days post diagnosis (DPD) was used instead. In parallel, the same samples were measured with a commercial ELISA (EuroImmun S1 ELISA; Fig. 2*D*). We also analyzed NIA and ELISA results split into samples obtained on or before 20 d post onset of symptoms and samples obtained after 20 d post onset when antibody responses are usually fully developed (Fig. 2 *A* and *D*). We calculated specificity and sensitivity using the NIA and ELISA results. For all specificity and sensitivity calculations, we classified the samples into condition-positive (sera from RT-PCR positive individuals) and condition-negative (prepandemic sera) groups. When all samples were used, we obtained a 100% specificity and 98% sensitivity for NIA, and a 99% specificity and 88% sensitivity for the commercial ELISA. When calculating the sensitivity values only for the subset of samples obtained after 20 d post onset of symptoms, the sensitivity was 98% for both NIA and the commercial ELISA. When calculating the sensitivity only for the subset of samples obtained on or before 20 d post onset, the sensitivity values were 98% for NIA and 85% for the commercial ELISA (Fig. 2*D*). As several SARS-CoV-2 antibody assays are now available on the market, we tested four additional commercially available assays to detect anti–SARS-CoV-2 IgG antibodies in a subset (*n* = 138) of the same condition-positive COVID-19 patient serum samples tested above. For samples collected after 20 d post onset, these assays returned sensitivity values in the range of 74 to 98% and, for samples collected less than 20 d post onset, returned sensitivity values ranging from 66 to 89% (*SI Appendix*, Fig. S3). These results indicate that NIA is a robust method for detecting SARS-CoV-2 antibodies in human serum samples.

On NIA, each sample dilution was tested in triplicate, enabling us to test up to 336 different samples or sample dilutions on a single device. In order to determine whether it would be possible to further increase throughput by reducing the number of replicates, we randomly selected one or two of the three 1:8 dilution replicates and calculated ROC curves, specificity, and sensitivity (*SI Appendix*, Fig. S4). We found that using duplicates only slightly reduced sensitivity from 98 to 97%, whereas using a single measurement lowered sensitivity to 95%, all the while retaining a 100% specificity. Given that using duplicate measurements led to only a minimal change in sensitivity, it will be possible to increase throughput to 512 samples analyzed in duplicate per device. Additional gains in throughput are theoretically possible by scaling the device itself, with MITOMI devices containing up to 4,160 unit cells having been demonstrated in the past (21).

To assess device-to-device reproducibility, we tested all serum samples (1:8 dilution) on two separate devices, which resulted in a correlation of $R^{2}=0.98$ (*SI Appendix*, Fig. S5*A*). We then correlated the measurements from one of those devices to the measurements made on six other devices and again observed a good correlation of $R^{2}=0.95$ (*SI Appendix*, Fig. S5*B*). In the first case, the same sample solution was used for spotting two devices, whereas, in the second case, the two measurements being compared came from separately prepared patient sample dilutions. Additionally, we compared the anti–IgG-PE signal measured for a reference serum dilution series on three separate devices and computed an average coefficient of variation (CV) equal to 11.7% for the dilutions measured (*SI Appendix*, Fig. S5 *C* and *D*).

To explore the technical capabilities of NIA, we determine whether quantitative antibody titers could be obtained. We fit data from the full dilution series to a saturation binding curve model, enabling us to extract the dilution equivalent to the half-maximum signal for each serum sample ($EC_{50}$) (Fig. 2*F* and *SI Appendix*, Fig. S6). Using the calculated $1/EC_{50}$ value, we again analyzed the NIA results, resulting in a specificity and sensitivity of 100% and 98%, respectively, showing that the use of dilutions did not further increase the sensitivity (Fig. 2*G*). Lastly, we compared the $1/EC_{50}$ values to values obtained from a single 1:8 dilution and found an excellent correspondence between those two measurements (Fig. 2*H*), indicating that a single measurement could be used to obtain information on antibody concentration. Only at very high antibody titers does the signal saturate for the 1:8 dilution. Using two measurements of a 1:8 and one slightly higher dilution could therefore maximize throughput, specificity, and sensitivity of NIA, and return accurate antibody titer values.

### Ultralow-Volume Whole Blood Collection and Analysis.

**Fig. 3.**
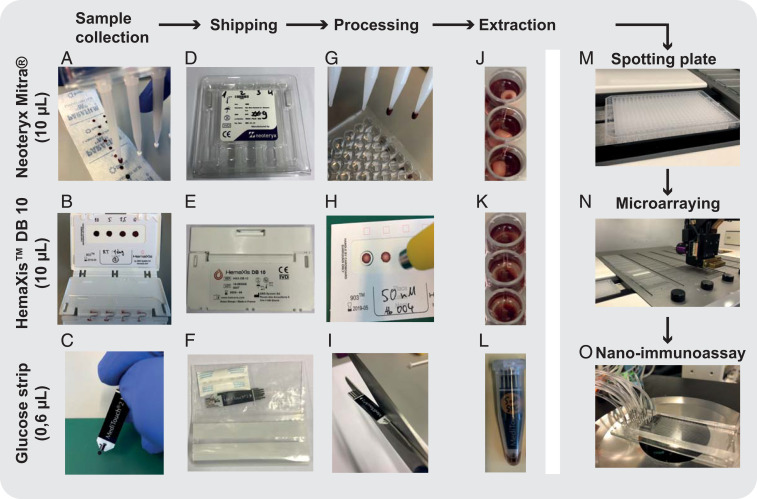
Ultralow-volume whole blood sampling and processing. Three devices were tested for ultralow-volume whole blood sampling and extraction: Neoteryx Mitra^®^, DBS System SA HemaXis™DB10, and glucose test strips; 10 μL of whole blood is collected by the (*A*) Mitra^®^d and (*B*) HemaXis™DB10 devices, and (*C*) 0.6 μL of whole blood is collected by the blood glucose test strip. (*D*–*F*) Blood samples are dried, allowing the devices to be shipped under ambient conditions by regular mail. (*G*–*I*) The devices are processed upon arrival at the laboratory. (*G*) Mitra^®^ tips are removed and placed in a 96-well plate; (*H*) HemaXis™DB10 cards are punched, and the filter discs are placed in a 96-well plate; and (*I*) the glucose test strip is cut to size and placed in an Eppendorf tube. (*J*–*L*) Blood samples are extracted in a buffer solution by overnight incubation at 4 °C, followed by (*M*) transfer to a spotting plate. Samples are then (*N*) microarrayed and (*O*) analyzed with the NIA device.

The NIA platform proved to be highly specific and sensitive when applied to serum samples. We recognized that use of serum samples limits the applicability of our and other methods to samples collected by venipuncture, which has to be performed by trained personnel in hospitals or point-of-care settings. Venipuncture requires large volumes of blood in the 5-mL range, considerable downstream sample processing, and a cold chain from point of sample collection to point of analysis. Large-scale seroprevalence studies are thus challenging to conduct, especially when including remote or large geographic areas. Establishing a large-scale, low-cost, and widely accessible serology platform necessitates the development of venipuncture alternatives. For this reason, we developed a sample collection and processing pipeline that enables us to carry out NIA with ultralow-volume, dried whole blood samples obtainable by a simple finger prick. We tested three different methods to collect, ship, process, extract, and analyze dried whole blood samples (Fig. 3). We tested two commercially available devices that collect defined 10-μL volumes of whole blood: Neoteryx Mitra^®^ and DBS System SA HemaXis™DB10. We also explored the possibility of repurposing existing blood glucose test strips (Medisana, MediTouch 2) for whole blood collection and shipping. The glucose test strips collect 0.6 μL of whole blood, which is approximately 20 times less than what is required by the commercial devices and 10,000 times less blood than obtained by venipuncture. Furthermore, blood glucose test strips are cheap at less than 0.5 USD per strip and widely available, potentially avoiding any supply bottlenecks during a pandemic. The two commercial devices, by comparison, cost 5 USD to 10 USD, or 1.25 USD to 2.5 USD per sample.

**Fig. 4.**
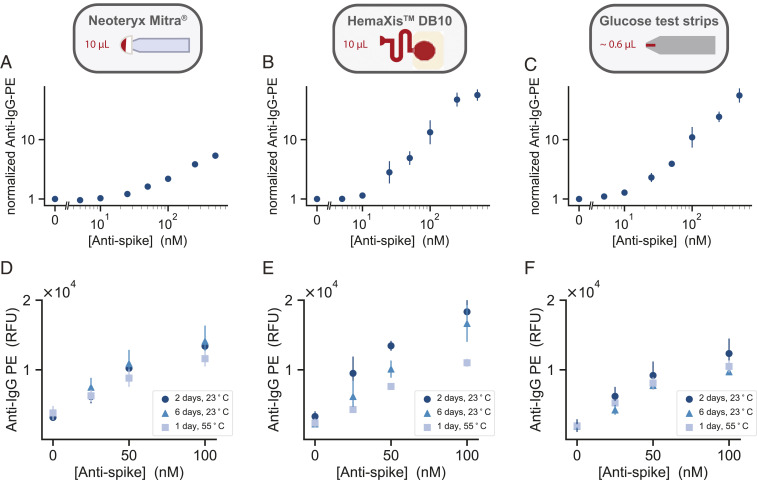
Ultralow-volume dried blood method characterization. The figure is organized into three columns, each corresponding to the respective sampling method: (*A* and *D*) Neoteryx Mitra^®^, (*B* and *E*) HemaXis™DB10, and (*C* and *F*) glucose test strips. (*A*–*C*) Normalized NIA anti–IgG-PE signal versus concentration of anti-spike IgG present in dried whole blood samples collected with each of the three sampling methods. Data points represent means ± SD (*n* = 4). The normalized signal is calculated by dividing the mean value for a given anti-spike concentration by the negative control mean value for 0 nM anti-spike. (*D*–*F*) Sample stability testing. NIA anti–IgG-PE measurements versus anti-spike IgG concentration. Blood samples were dried and then stored on each collection device for 1 d or 6 d at 23 °C, and 1 d at 55 °C.

We evaluated the three collection methods by spiking human whole blood with different concentrations of anti-spike IgG and collected the blood with each collection device. We then extracted the dried blood from each device and spotted the samples for NIA analysis. We first tested an aqueous ethylenediaminetetraacetic acid (EDTA) solution and sonication for extraction (22), but the use of this buffer resulted in large and inconsistent spots during microarraying. We then tested phosphate-buffered saline (PBS, 1% BSA) or PBS (1% BSA, 0.5% Tween-20) (23) extraction buffers with overnight incubation at 4°C and found that the addition of 0.5% Tween-20 greatly improved the assay. With this optimized sample extraction workflow, we were able to quantitate anti-spike IgG from the dried blood samples (Fig. 4 *A*–*C* and *SI Appendix*, Fig. S7).

To assess the variability of each sampling method, we collected whole blood spiked with anti-spike IgG three separate times and compared the on-chip antibody signals (*SI Appendix*, Fig. S8 *A*–*C*). We calculated the coefficient of variation for the technical repeats for each anti-spike concentration tested and found that it did not exceed 15% for any of the three collection methods, with an average CV of 5.7%, 7.7%, and 9.2% for Mitra^®^, HemaXis™DB10, and the glucose test strips, respectively (*SI Appendix*, Fig. S8*D*). Although, there may be further variability introduced depending on how each individual collects his or her own blood sample, each device uses a hard-coded method to collect a specific volume of blood that leads to low variability for NIA antibody detection.

As we expect the use of these blood sampling devices to be decentralized, followed by shipping with regular mail to a central laboratory for analysis, an important factor to assess was sample stability. To determine stability of anti-spike IgG antibodies, we allowed the blood to dry on each device, followed by storage for 2 and 6 d at room temperature (23°C) before extraction and testing (Fig. 4 *D*–*F*). Furthermore, depending on the climatic conditions, samples sent by post may be subject to higher temperatures; therefore, we also stored the devices for 1 d at 55°C. For the Mitra^®^ device, we observed very little sample degradation after 6 d of storage at room temperature and slight sample degradation when stored at 55°C for 1 d. Very similar results were obtained for the glucose test strips. The HemaXis^TM^DB10 devices were the most sensitive to prolonged and high-temperature storage, but still resulted in sufficient signal for quantitation.

Having established that ultralow-volume dried blood samples can be analyzed on the NIA platform, we tested the method with ultralow-volume whole blood patient samples collected with each of the three collection methods. As a surrogate for capillary blood, we used 36 EDTA whole blood samples from 21 RT-PCR–confirmed COVID-19 patients and 15 presumed negative patients hospitalized for other reasons that served as negative controls. We collected whole blood samples in Geneva followed by shipping via regular mail to Lausanne for analysis. All positive samples are early seroconverts obtained within 14 d post diagnosis, and thus even standard, large-volume serum samples are challenging to analyze (Fig. 2*C*). For reference measurements, we prepared plasma from EDTA whole blood samples and performed the commercial EuroImmun S1 ELISA assays on the same patient samples.

**Fig. 5.**
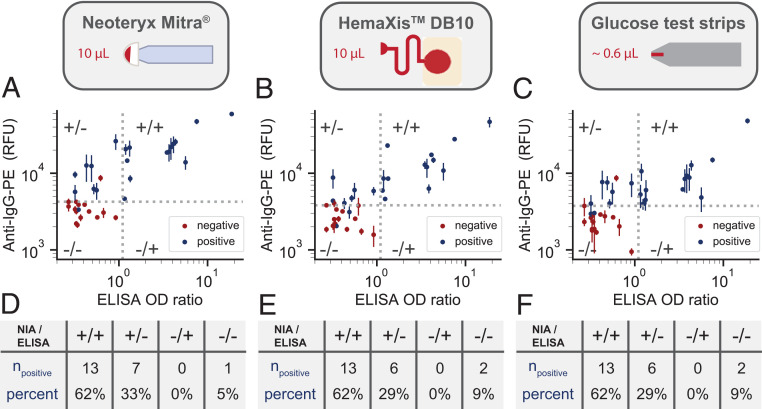
Ultralow-volume patient sample collection and analysis. (*A*–*C*) NIA anti–IgG-PE signal versus ELISA optical density (OD) ratio for whole blood patient samples collected using each of the three sampling methods: Neoteryx Mitra^®^, DBS System SA HemaXis™DB10, and glucose test strips. Data points are colored either blue or red, corresponding to whether the samples were presumed positive or negative. The vertical dashed line represents the positive–negative cutoff for ELISA, and the horizontal dashed line represents the chosen cutoff for the NIA measurements set equal to the second-highest negative measurement. Data points represent means ± SD (*n* = 4). (*D*–*F*) Number of blue data points for each quadrant in *A*–*C*, respectively, along with the percentage of positive data points per quadrant.

To assess the technical properties of combining dried blood sampling with downstream NIA analysis, we directly compared results obtained by ELISA performed on standard large-volume serum samples to NIA measurements performed on ultralow-volume dried whole blood samples collected with the three collection methods and processed as described above (Fig. 5). ELISA detected SARS-CoV-2–specific antibodies in 62% of all COVID-19 patient samples and found no detectable antibodies in any of the 15 presumed negative samples. For the three sampling methods, we set the threshold between positive and negative cells to the intensity of the second-highest negative sample. All three methods identified the same 62% anti–SARS-CoV-2 IgG positive samples as the reference ELISA, but the Mitra^®^ method was able to detect antibodies in 33% additional RT-PCR positive samples, and the HemaXis™DB10 and glucose strip methods were able to detect an additional 29%. This proof of concept study demonstrates that ultralow-volume whole blood samples can be collected and analyzed on the NIA platform, and that even difficult-to-quantitate samples obtained within the first 14 d post onset of symptoms could be analyzed with this approach.

## Discussion

**Fig. 6.**
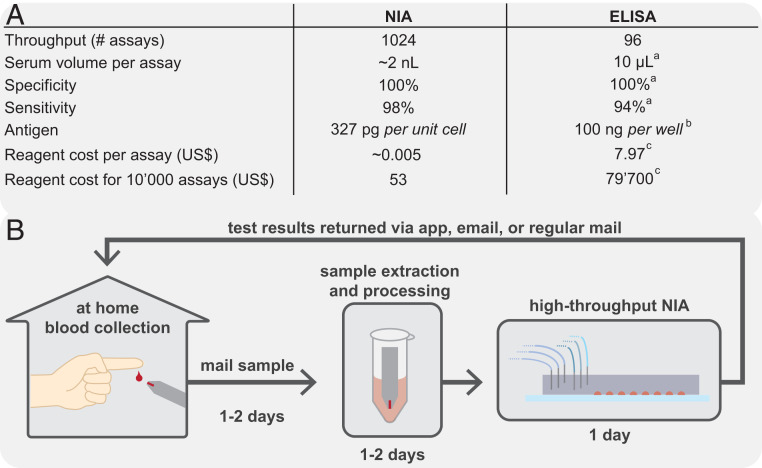
NIA performance table and conceptual home-based sample collection and centralized NIA analysis. (*A*) Comparison between the high-throughput NIA platform and standard ELISA. Sensitivity and specificity values are based on serum sample analysis for both NIA and ELISA. Entries with superscript a are based on EuroImmun ELISA kit (EI 2606-9601) (29), those with superscript b are based on ref. 23, and those with superscript c are priced based on Abcam ELISA kit (ab274342). (*B*) An NIA-based diagnostic workflow that uses decentralized ultralow-volume whole blood sample collection, shipping via regular mail, and centralized sample processing and analysis on a high-throughput NIA platform. Test results are analyzed and interpreted and then reported back via mobile app, email, or regular mail.

We developed and validated a high-throughput NIA device capable of analyzing 512 to 1,024 samples in parallel. Detecting the presence of SARS-CoV-2 anti-spike IgG antibodies with a 512-sample throughput per device, the method achieved a specificity of 100% and a sensitivity of 98% based on the analysis of serum samples from 155 positive SARS-CoV-2–infected and 134 negative individuals (Fig. 6*A*). When analyzing 1,024 samples per device, the method achieved a sensitivity of 95%. These results indicate that an accurate binary classification of serum samples can be achieved with NIA.

The method developed here is applicable to the large-scale characterization of serum samples collected as part of epidemiological studies, identifying donors for plasma therapy, and vaccine trial support. A single researcher can achieve a throughput of one or two devices, or 512 to 1,024 samples per day (analyzed in duplicate) in a small research laboratory not dedicated or equipped for high-throughput molecular diagnostics. Due to increases in efficiency, a small team of three can likely achieve a throughput of six devices or 3,072 samples per day (analyzed in duplicate). By comparison, the number of RT-PCR tests performed in all of Switzerland ranged from 6,000 to 18,000 tests per day between April and September 2020. Consumables, reagent consumption, and associated costs are negligible with NIA, which is an important consideration when compared to the high reagent cost of ELISAs and when considering potential reagent shortages that may be encountered during critical phases in a global pandemic.

To implement the method, laboratories require a commercially available contact microarrayer, and the ability to fabricate masks, molds, and PDMS microfluidic devices. Instruments required for photolithographic fabrication of masks and molds are available at most major research universities, and mask fabrication can be outsourced to service-based companies. PDMS microfluidic device fabrication can be conducted in a standard research laboratory with low-cost equipment: a spin coater, 80°C oven, and stereomicroscope. To run the microfluidic devices, standard pressure regulators, pressure gauges, and manual or solenoid valves are used. Device readout is performed on a standard epifluorescence microscope equipped with an automated stage.

Platform capabilities could be further expanded in the near term. We previously demonstrated that the platform can be used to perform multiplexed analysis, allowing four or more biomarkers to be tested for each sample (17, 18, 24). This would potentially enable the analysis of multiple antigens, cytokines, or inflammatory markers to provide insights into prior virus exposures and the response to infection. It should also be possible to detect IgA and IgM isotypes by using detection antibodies specific for these isotypes and to do so in a multiplexed format in which all three isotypes, IgG, IgA, and IgM, are measured per sample. We also previously demonstrated that kinetic rate measurements can be performed in high throughput with this method (25) and that digital ELISAs can be performed using the same technology in instances where lower limits of detection may be required (11, 26).

To enable large-scale studies, we placed specific emphasis on the development of simple, low-cost, and ultralow-volume sample collection strategies and integration of these workflows with the NIA platform. We characterized three blood sampling devices and tested all of them with patient samples. Two of these are commercially available devices for the express purpose of collecting small-volume capillary blood samples: Mitra^®^ from Neoteryx and HemaXis™DB10 from DBS System SA. We also explored the possibility of repurposing low-cost and readily available blood glucose test strips for blood sample collection and shipment. Mitra and DB10 allow the collection of a minimal volume of 10 μL, whereas the blood glucose test strip collects as little as 0.6 μL of whole blood. Such small volumes allow untrained personnel to finger-prick and collect whole blood, eliminating the need for phlebotomists and the inconvenience of visiting a hospital or point-of-care location. The collected blood is allowed to dry on the devices, and we showed that these dried blood samples can be analyzed after 6 d of storage at ambient temperatures, eliminating the need for a cold chain.

The combination of a high-throughput, highly specific and sensitive NIA and the ability to analyze minute volumes of dried blood samples have enormous potential for SARS-CoV-2 serology, epidemiological studies, vaccine trial, and therapeutic development support. Further areas of use could be large-scale seroprevalence studies in low- and middle-income countries (27) without sufficient in-country laboratory capacity, by sending specimens by international mail. Especially in the current SARS-CoV-2 pandemic, population-based seroprevalence studies could elucidate some urgent questions, such as the impact of COVID-19 in Africa (28).

New technology and method developments such as those reported here will make it possible to overcome the current centralized molecular diagnostics paradigm, which is focused on hospitals and point-of-care settings rather than on patients and individuals seeking simple, affordable, and convenient molecular diagnostics. The need to visit a clinic is an inconvenience for everyone and can be an insurmountable obstacle for many. The requirement for venipuncture blood collection, sample pretreatment, and costly ELISAs prohibits broad testing and contributes to high health care costs.

In the future, NIA could make it possible for individuals to purchase a simple blood sampling kit containing a lancet, a blood sampling device, and a return mail envelope, at a local pharmacy or supermarket (Fig. 6*B*). The kit can be used easily and conveniently in the privacy of one’s own home, where a simple finger prick is made, and the blood is collected with the device. The device with the collected blood can then be sent, without special biosafety requirements, by regular mail to a central laboratory which analyzes the blood sample for one or more biomarkers, interprets the data, and returns the test results to the individual via smart phone, email, or regular mail. Furthermore, each blood sample is sufficient to conduct many molecular diagnostic assays with NIA. The whole process from kit purchase to results could take less than a week, which is fast enough for a vast majority of tests that are not particularly time critical. Decentralized and simple sample collection coupled with centralized, next-generation, high-performance molecular tests will broaden access to molecular diagnostics, and increase the use of testing. During a global pandemic, such technologies could enable the collection of critical epidemiological data, providing instrumental data for vaccine development and vaccine rollout.

## Materials and Methods

Full details are given in *SI Appendix*. High-throughput NIA was conducted using a PDMS microfluidic device. Patient serum or extracted whole blood samples were spotted onto an epoxy-coated glass slide using a microarray robot. All samples were analyzed for the presence of anti-spike IgG antibodies on-chip. In parallel, commercial IgG ELISA assays were performed for all samples according to the manufacturer’s instructions. All samples were collected according to the local ethical guidelines, and ethical approval was waived by the ethics committee of the University Hospital of Geneva (HUG), that approves usage of leftover patient serum collected for diagnostic purposes, in accordance with Swiss Regulations on human research. Patients signed the general informed consent form of the HUG, which states that any leftover material can be used for research purposes.

## Supplementary Material

Supplementary File

Supplementary File

Supplementary File

## Data Availability

All study data are included in the article, *SI Appendix*, and Datasets S1 and S2.
