# Supplementary material for: A high-throughput microfluidic nanoimmunoassay for detecting anti–SARS-CoV-2 antibodies in serum or ultralow-volume blood samples
Source: Proc Natl Acad Sci U S A. 2021 Apr 16;118(18):e2025289118. doi: 10.1073/pnas.2025289118 (PMC8106336; doi:10.1073/pnas.2025289118)
Supplement: Supplementary File [file pnas.2025289118.sapp.pdf]

www.pnas.org

Supplementary Information for

A high-throughput microfluidic nano-immunoassay for detecting anti-SARS-CoV-2 antibodies in serum or ultra-low volume blood samples

Zoe Swank, Grégoire Michielin, Hon Ming Yip, Patrick Cohen, Diego O. Andrey, Nicolas Vuilleumier, Laurent Kaiser, Isabella Eckerle, Benjamin Meyer and Sebastian J. Maerkl

Isabella Eckerle, Benjamin Meyer and Sebastian J. Maerkl.
E-mail: isabella.eckerle@hcuge.ch, benjamin.meyer@unige.ch and sebastian.maerkl@epfl.ch

This PDF file includes:

Supplementary text
Figs. S1 to S9 (not allowed for Brief Reports)
Legends for Dataset S1 to S2
SI References

Other supplementary materials for this manuscript include the following:

Datasets S1 to S2

Supporting Information Text

Microfluidic chip fabrication

The designs for the flow and control layer of the device were drawn with AutoCAD software and are available for download (lbnc.epfl.ch), we then used standard photolithography to fabricate the molds for each layer. Three replicates of the design are fitted onto a 4 inch silicon wafer so that three devices can be made in a single fabrication process. SU-8 negative photoresist was used to create the control channel features (GM 1070, Gersteltec Sarl) with a height of 30 μm, while AZ 10XT-60 positive photoresist (Microchemicals GmbH) was used to generate flow channel features with a height of 15 μm. After development, the flow layer mold was annealed at 180°C in a convection oven for two hours to obtain rounded features. Afterwards each of the wafers was treated with TMCS (trimethylchlorosilane) and coated with PDMS (Sylgard 184, Dow Corning). For the control layer ~50 g of PDMS with an elastomer to crosslinker ratio of 5:1 was prepared, whereas for the flow layer a 20:1 ratio of elastomer to crosslinker was spin coated at 400 rcf to yield a height of ~50 μm. Both PDMS coated wafers were then partially cured for 20 minutes at 80°C, after which devices from the control layer were cut out and the inlets for each control line were punched (OD = 889 mm) using a precision manual-punching machine (Syneo, USA). Each control layer is then aligned onto the flow layer by hand using a Nikon stereo microscope. The aligned devices were then placed at 80°C for 90 minutes, allowing the two layers to bond together so that the entire device can then be cut and removed from the flow wafer. After that, the flow layer inlets were punched.

Immunoassay reagents

For our on-chip immunoassay we used biotinylated mouse anti-His antibodies (Qiagen, 34440) to immobilize His-tagged SARS-CoV-2 antigens on the surface of our assay chambers. The prefusion ectodomain of the SARS-CoV-2 spike glycoprotein (the construct was a generous gift from Prof. Jason McLellan, University of Texas, Austin (1)) was transiently transfected into suspension-adapted HEK293 cells (Thermo Fisher) with PEI MAX (Transfection grade linear polyethylenimine hydrochloride, Polysciences) in Excell293 medium. Incubation with agitation was performed at 37°C and 4.5% CO2 for 5 days. The clarified supernatant was loaded onto Fastback Ni2+ Advance resin column (Protein Ark) eluted with 500 mM imidazole, pH 7.5 in PBS. For proof-of-concept experiments, chimeric anti-spike antibodies were purchased from Sino Biological (40150-D002, 40150-D003, 40150-D004, 40150-D005). We spiked the chimeric anti-spike antibodies into human serum (Sigma-Aldrich, H4522) and whole blood (ZenBio, SER-WB). For detecting human IgG, we used PE labeled goat anti-Human-IgG (Abcam, ab131612).

Sample preparation

Serum. Serum samples were collected from 155 RT-PCR confirmed COVID-19 patients and 134 negative control sera obtained in 2013/14 and 2018 before the start of the pandemic (including 50 children). We used days post onset of symptoms (dpos) according to patient history or days post diagnosis (dpd) in case dpos was unknown. All samples were stored at -20°C until analysis. In order to handle patient serum samples in a Biosafety Level 1 laboratory, patient serum samples were heat treated at 56°C for 30 minutes and Triton X-100 (Fisher Scientific) was added to the samples to a final concentration of 1%. In order to optimize spotting parameters and the on-chip immunoassay, proof-of-concept experiments involving human serum spiked with chimeric anti-spike antibodies were also carried out with the addition of Triton X-100. For dilution series experiments, patient serum samples were diluted in a PBS solution containing 2% BSA. Additionally, 10 μM fluorescein isothiocyanate(FITC)-dextran (10 kDa) was added as a tracer to each sample in order to assess whether similar volumes of serum samples were spotted (Fig. S9).

Whole and dried blood. Human Whole Blood - Frozen, 10ml was obtained from AMS Biotechnology (Europe). Anti-spike antibodies (Sino Biological) were added to whole blood to the desired concentration from a stock solution of 2 μM. To simulate a fingerprick collection, 15-20 μL of whole blood samples were pipetted on parafilm and then collected on the sampling device where 10 μL were collected with HemaXis™ DB10 (DBS System SA) or Mitra® Clamshell (Neoteryx). The samples were dried and then stored in their original container without further protection. Samples were kept at room temperature for 1, 2 or 6 days, or in a 55°C oven for 1 day. The dried blood stored on HemaXis™ filter paper were cut using an 8 mm biopsy puncher and scalpel and the tip of the Mitra® devices were removed with tweezers. The samples were placed in a 96-well plate and filled with 200 μL of cold extraction buffer (1xPBS, 1% BSA, 0,5% Tween20) (2) and incubated overnight at 4°C with 300 rpm agitation. Around 150 μL per well of the supernatant was recovered and stored at -20°C until analysis. Alternatively, a small volume of blood (around 0.6 μL) was collected with a Meditouch 2 glucose test strip (Medisana), dried and stored in a box kept at room temperature for 1,2 or 6 days, or in a 55°C oven for 1 day. Extraction was performed by placing the strip at the bottom of a 1,5 mL tube filled with 30 μL of extraction buffer overnight at 4°C.

As a substitute for capillary blood taken from a fingerprick, we used leftover EDTA whole blood samples drawn from hospitalised COVID-19 patients at different time points post diagnosis for routine clinical laboratory analysis. EDTA whole blood samples were stored at 4°C for a maximum of seven days before they were applied to the dried blood collection devices. 20 μL of EDTA whole blood was pipetted on a parafilm sheet, and collected with a Mitra® device or glucose strip. For the HemaXis™ device, 10 μL of whole blood was directly pipetted on the filter card. The samples were dried for 30 minutes and placed in a plastic bag with silica gel before shipping. The samples were extracted as described above 5 days after collection and shipping, microarray spotted on a glass slide on day 6 and the nano-immunoassay chip was run on day 7. To determine

antibodies against SARS-CoV-2 S1 protein by Euroimmun S1 ELISA, EDTA whole blood samples where centrifuged for 5min at 1200x g at 4°C and 200 μL of plasma were stored at 4°C until analysis.

SARS-CoV-2 serological assays. Euroimmun S1 IgG ELISA (Euroimmun AG, Lübeck, Germany, # EI 2606-9601 G) was performed according to the manufacturers instructions and was run on a Dynex Agility (RUWAG Handels AG, Bettlach, Switzerland). OD ratios were calculated by dividing the OD450 of each sample by the OD450 of a calibrator that was run on each plate. Results equal or above OD ratio of 1.1 were considered positive. The LIAISON SARS-CoV-2 S1/S2 IgG ELISA was run on the LIAISONR XL analyzer, (Diasorin, Italy), the EDI Novel Coronavirus COVID-19 IgG ELISA (Epitope Diagnostics, USA) on the DSX analyzer (Dynex, Switzerland) and the Elecsys Anti-SARS-CoV-2 N (anti-N total antibodies) as well as the Elecsys Anti-SARS-CoV-2 S (anti-S1-RBD total antibodies) on the Cobas e801 analyzer (Roche Diagnostics, Switzerland) according to the manufacturers instructions.

Microarray spotting

25 μL of each sample were loaded into a 384 microwell plate (ArrayIt, MMP384). An MP3 microarray printing pin (Arrayit) was used to spot the samples onto an epoxy-coated glass slide using a QArray2 microarrayer (Genetix). The presence of Triton X-100 in the serum samples had a significant effect on the spot diameter. To account for this we increased the dimensions of the spotting chamber and set the inking and stamping time to 50 ms and 1 ms, respectively. In addition, it was critical that the ambient humidity was below ~42%, otherwise the spots would become too large and merge together. After spotting, the PDMS chip was aligned on top of the sample spots using a stereo microscope and bonded over night at 40 °C.

Running on-chip immunoassays

Control lines were filled with PBS, attached to the chip and pressurized at 145 kPa. The NIA device is a low-complexity device, and can therefore be either regulated with simple manual 3 way toggle switch valves or computer controlled solenoid valves (only needed in order to make the entire on-chip workflow fully automated). Control and flow pressures are set by standard pressure regulators using a building air supply as the pressure source and monitored with two pressure gauges, respectively. Detailed descriptions of a standard MITOMI setup (3) and more sophisticated computerized microfluidic control setups for controlling complex multi-layer microfluidic devices have been previously published (4, 5). While isolating the spotted sample with the neck valve closed, the lower half of the unit cells were patterned with BSA-biotin (Thermo Fisher, 29130) and neutrAvidin (Thermo Fisher, 3100). First BSA-biotin was flowed at a concentration of 2 mg/mL for 20 minutes, then neutrAvidin was flowed at a concentration of 1 mg/mL for 20 minutes. A flow pressure of 27-34 kPa was maintained for each of the flow steps. Afterwards the button valve was actuated and BSA-biotin was flowed for an additional 20 minutes. In between each of these steps a solution of 0.005% Tween 20 (Sigma, P1379) in PBS was flowed for 5 minutes to wash away any unbound material. After surface functionalization, a 1 μg/mL solution of biotinylated anti-His antibody in 2% BSA in PBS was flowed for 20 minutes. Next a 6.7 μg/mL solution of His-tagged SARS-CoV-2 spike protein in 2% BSA in PBS was flowed for 20 minutes. The spike protein solution contained 25% chicken serum (Sigma, C5405), which served to block the surface and reduce non-specific binding. Before each of these steps the solution was first flowed for 2 minutes with the button valves down, allowing the solution to flow evenly into the entire device. After flowing each solution for 20 minutes, a wash step was performed by flowing 0.005% Tween PBS for 5 minutes with the button valves down. Once the spike protein was attached to the surface, the serum spots were re-solubilized by opening the neck valve and allowing 0.005% Tween PBS to flow into the spotting chamber while the outlet valve was closed. The neck valve was then closed and 0.005% Tween PBS was flowed for 5 minutes to prevent cross-contamination of samples in neighboring unit cells. The sandwich valves were then closed and the neck valve was released to allow any antibodies present in the serum spot to diffuse into the assay chamber. After an incubation of 70 minutes the button valve was opened and a second incubation of 60 minutes was carried out, permitting any anti-spike antibodies to bind to the spike protein. Any unbound material could then be washed away by flowing 0.005% Tween PBS for 5 minutes with the button and neck valves closed. A 5.6 μg/mL solution of anti-IgG-PE was then flowed for 2 minutes with the buttons down, then for 10 minutes with the buttons up. The buttons were then closed and any unbound detection antibody was washed away by flowing 0.005% Tween PBS for 5 minutes. Each unit cell was then imaged with an exposure time of 300 ms using a Nikon ECLIPSE Ti microscope equipped with a LED Fluorescent Excitation System, a Cy3 filter set, and a Hamamatsu ORCA-Flash4.0 camera (C11440).

Data analysis

The detection antibody signal was quantified using a custom Python script. ROC analysis was performed using GraphPad Prism. Cutoff values for the NIA measurements were chosen by maximizing both the specificity and sensitivity values. EC_{50} values were determined by fitting the dilution series data (1:256 - 1:8) for each sample to a saturation binding curve, $y = \frac{B_{max}x}{x+EC_{50}}$. For all samples B_{max} was set to 65000.

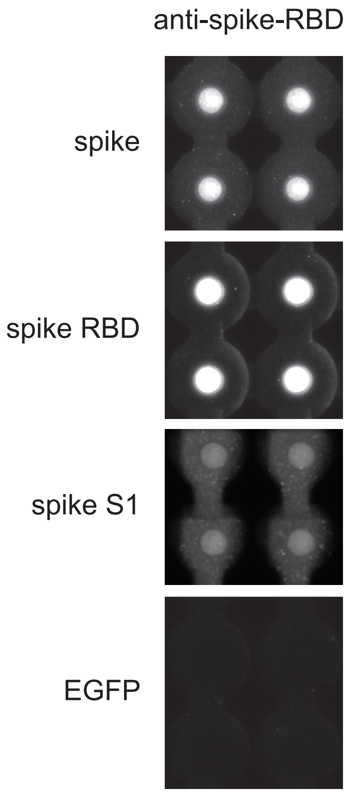

Fig. S1. Evaluation of SARS-CoV-2 antigens. NIA images showing anti-IgG-PE signals obtained when spike, RBD and S1 antigens in combination with an anti-S1 primary antibody were tested. EGFP served as a negative control.

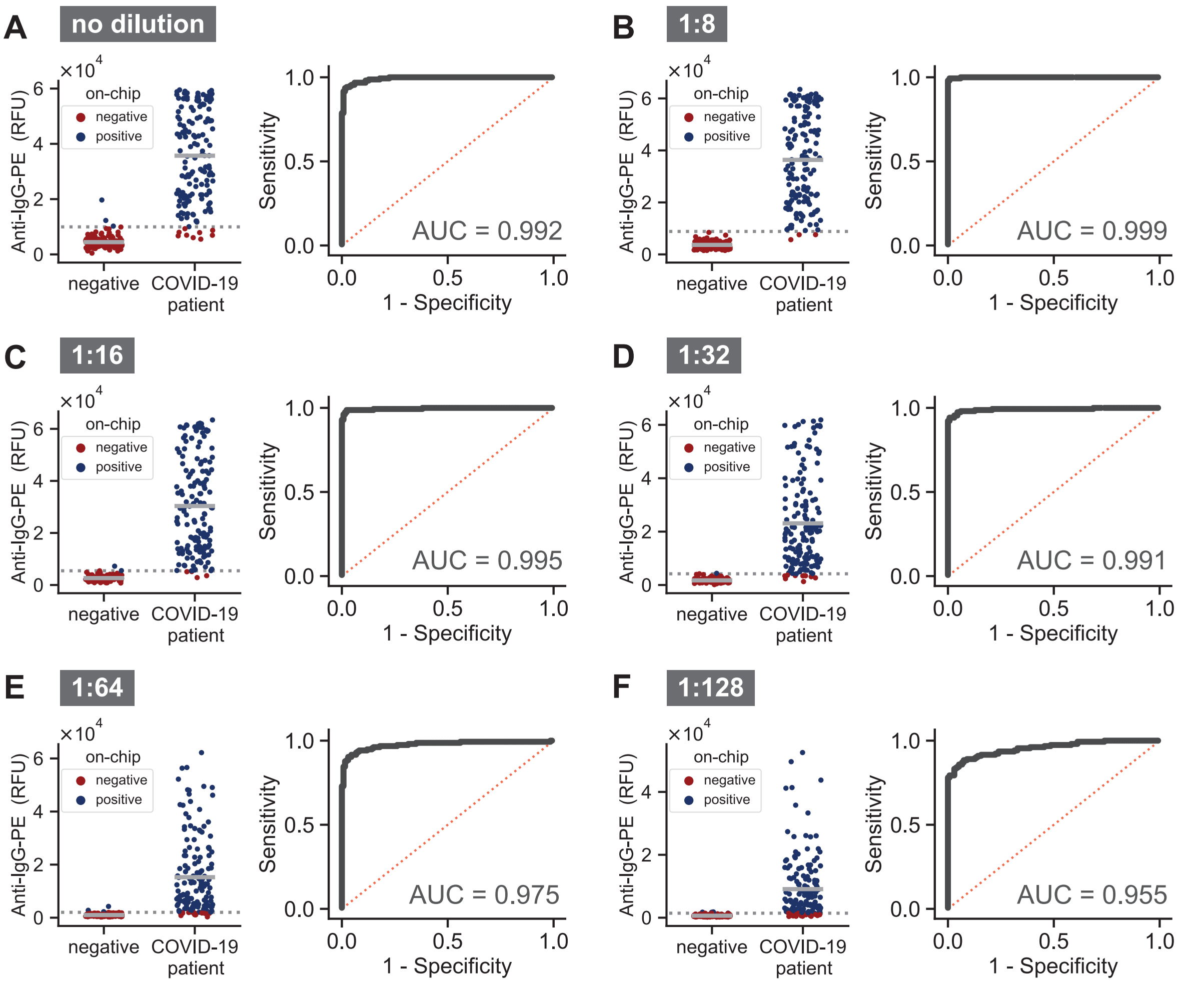

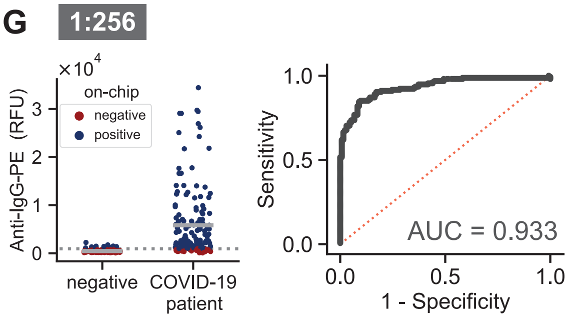

H

dilution	specificity	95% CI	sensitivity	95% CI
none	98%	93-99%	94%	89-96%
1:8	100%	97-100%	98%	94-99%
1:16	98%	94-99%	97%	93-98%
1:32	99%	95-99%	94%	89-96%
1:64	97%	92-98%	90%	84-94%
1:128	94%	89-97%	85%	79-90%
1:256	91%	85-95%	81%	74-86%

Fig. S2. NIA measurements for a range of sample dilutions. (A-G) NIA measurments for different dilutions of patient serum samples categorized according to whether the sample was pre-pandemic negative or from COVID-19 patients. Data points represent mean values ($n = 3$). Corresponding ROC curves are shown to the right of the plotted data. **(H)** Specificity and sensitivity values calculated for each dilution according the dashed cutoff line shown in plots **A-G**.

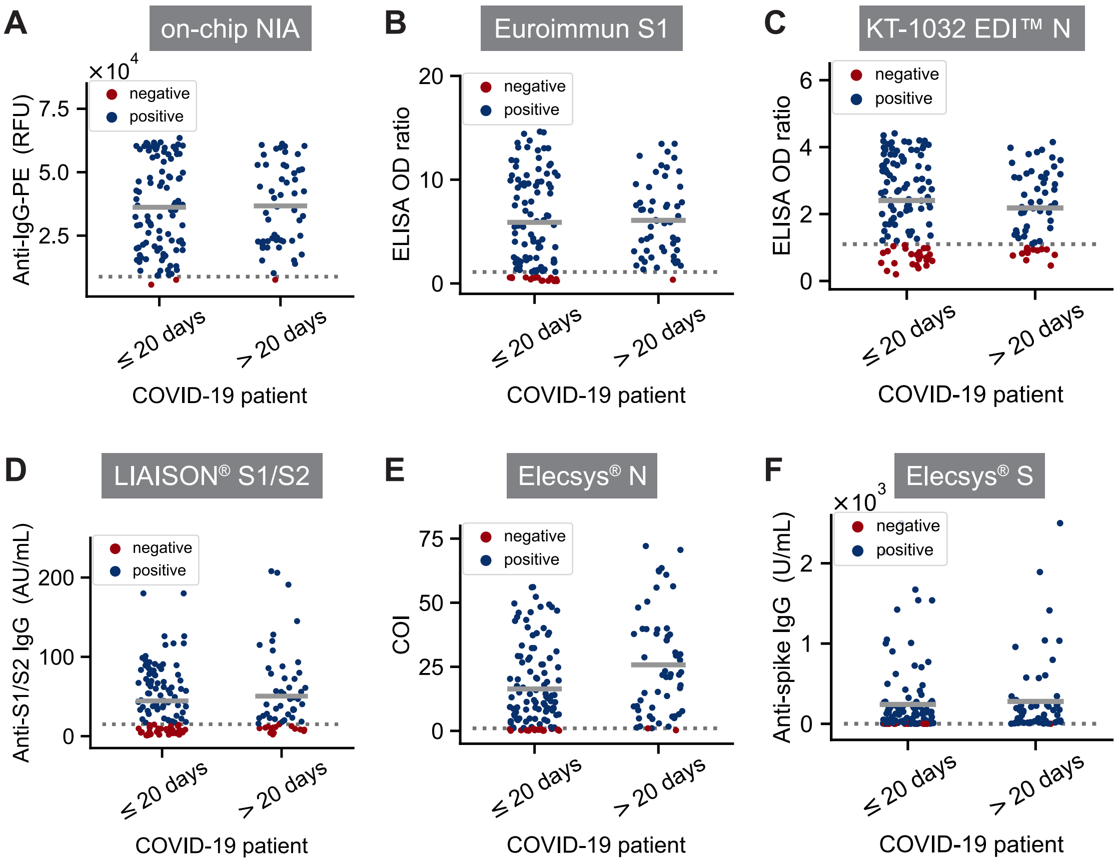

G

Sensitivity

	on-chip NIA	Euroimmun S1	KT-1032 EDI™ N	LIAISON® S1/S2	Elecsys® N	Elecsys® S
≤ 20 days	98%	85%	77%	66%	83%	89%
> 20 days	98%	98%	79%	74%	97%	98%
n_{total}	155	155	138	138	138	138

Fig. S3. Comparison of NIA with commercial assays. (A-F) Levels of anti-SARS-CoV-2 IgG antibodies present in serum collected from COVID-19 patients $\leq$20 days or $>$20 days post onset. **(G)** Sensitivity values calculated for each assay according to the dashed cutoff line shown in plots **A-F**. The manufacturer recommended cutoffs were used for the commercial assays. The total number of patient serum samples tested for each assay is listed below the sensitivity values.

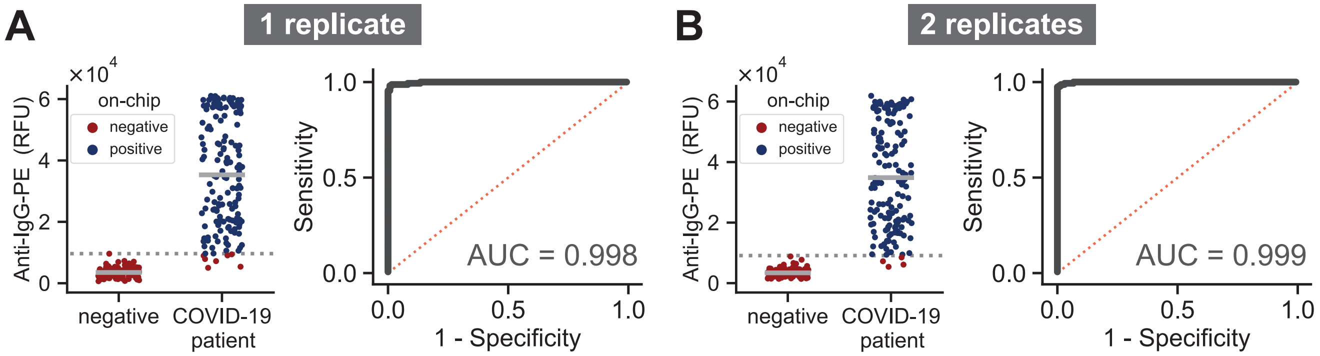

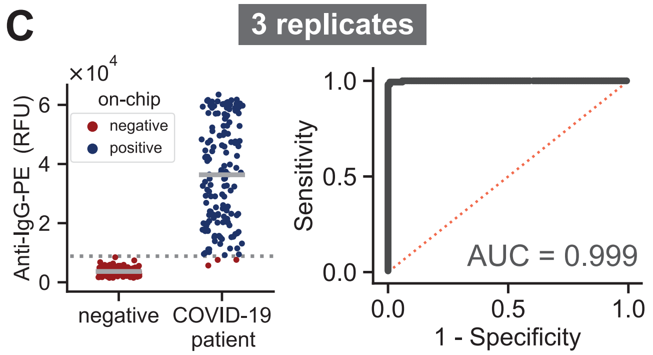

D

replicates	specificity	95% CI	sensitivity	95% CI
1	100%	97-100%	95%	90-97%
2	100%	97-100%	97%	93-98%
3	100%	97-100%	98%	94-99%

Fig. S4. NIA replicates. (A-C) Mean anti-IgG-PE signal for one, two or three on-chip replicates shown for a 1:8 serum dilution, along with the corresponding ROC curves. **(D)** Specificity and sensitivity values calculated according number of on-chip replicates and based on the dashed cutoff line shown in plots **A-C**.

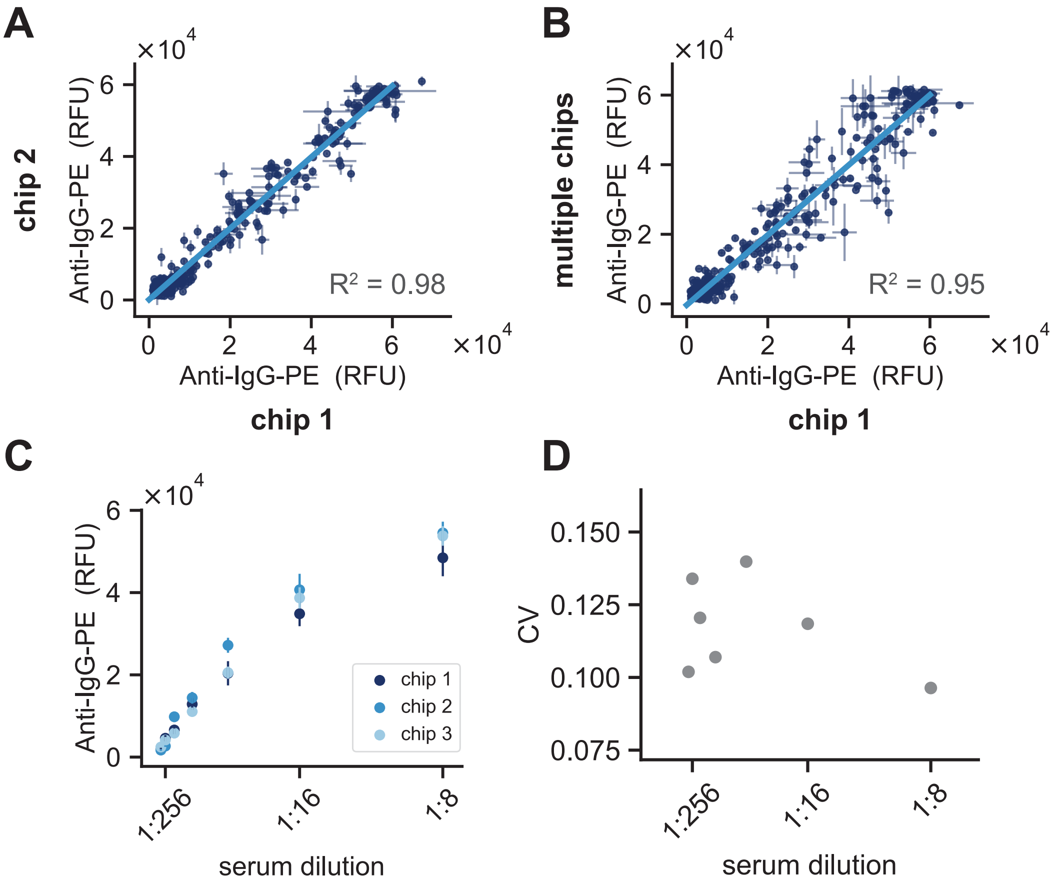

Fig. S5. Device-to-device variation. (A) Correlation of anti-IgG-PE signals obtained from two separate chips that were prepared using the same 1:8 serum sample dilutions. **(B)** Anti-IgG-PE measurements collected from a total of 6 chips versus measurements for the same samples collected on a single chip. Sample dilutions were prepared separately for each of the chips. **(C)** NIA measurements for a reference serum dilution series measured on three separate chips. **(D)** Coefficient of variation calculated for the three measurements of each reference serum dilution.

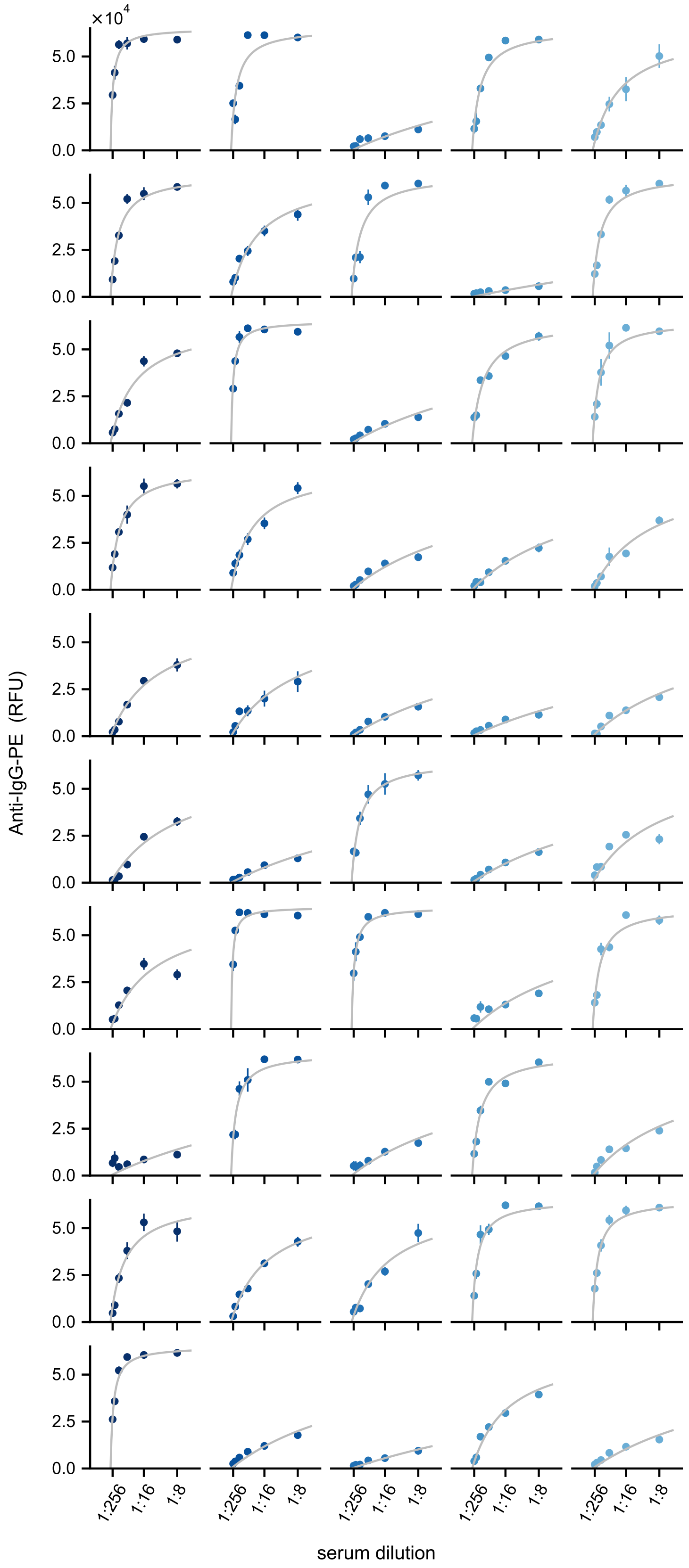

Fig. S6. Complete serum dilution data. Data points are colored blue or red corresponding to dilutions from negative or positive patient serum samples, respectively.

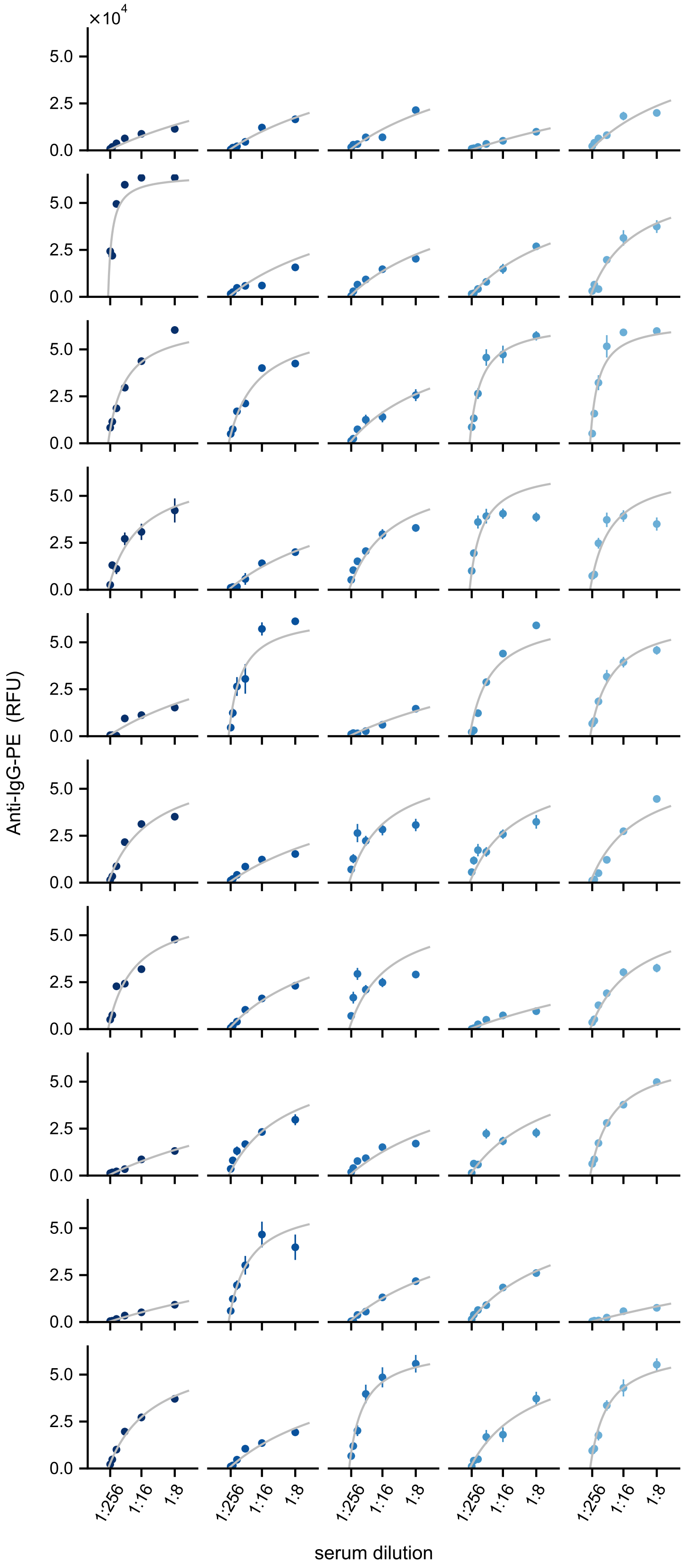

Fig. S6. Complete serum dilution data.

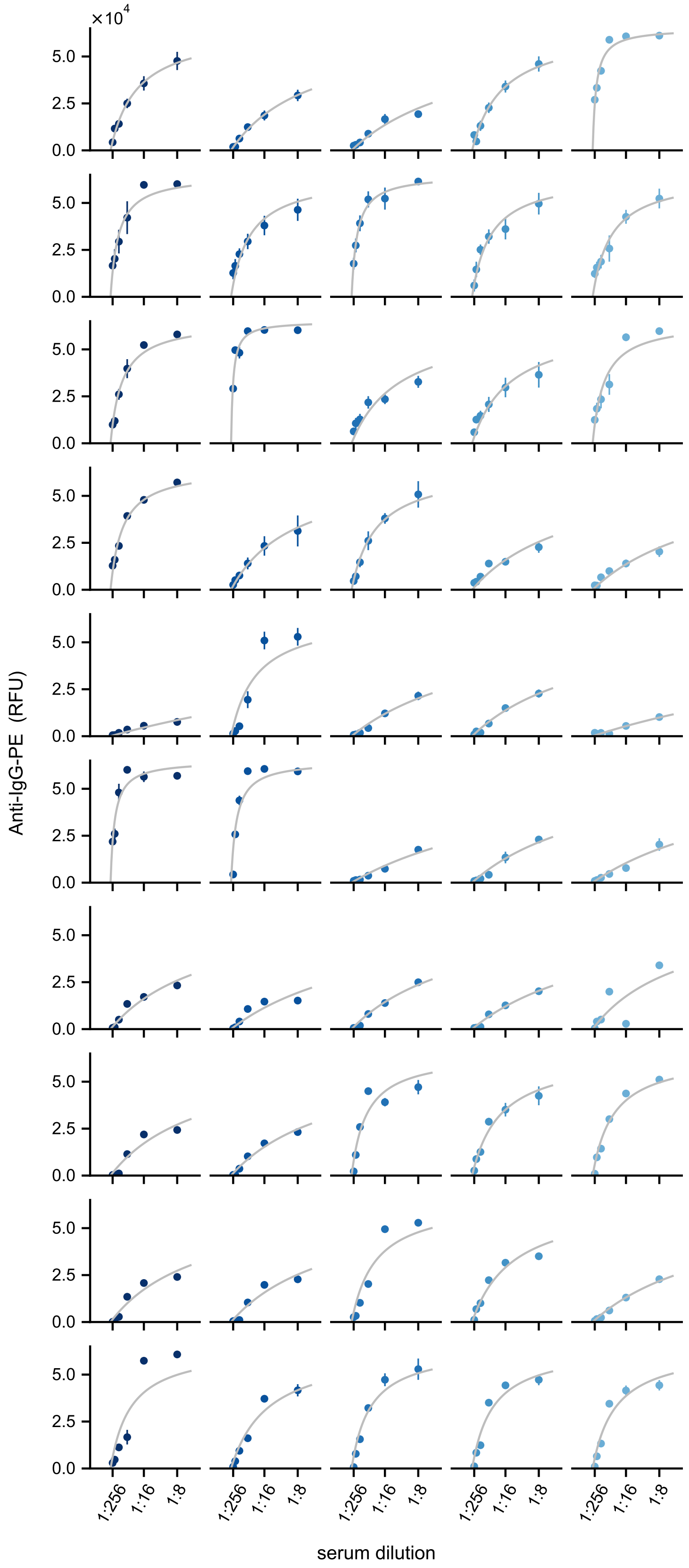

Fig. S6. Complete serum dilution data.

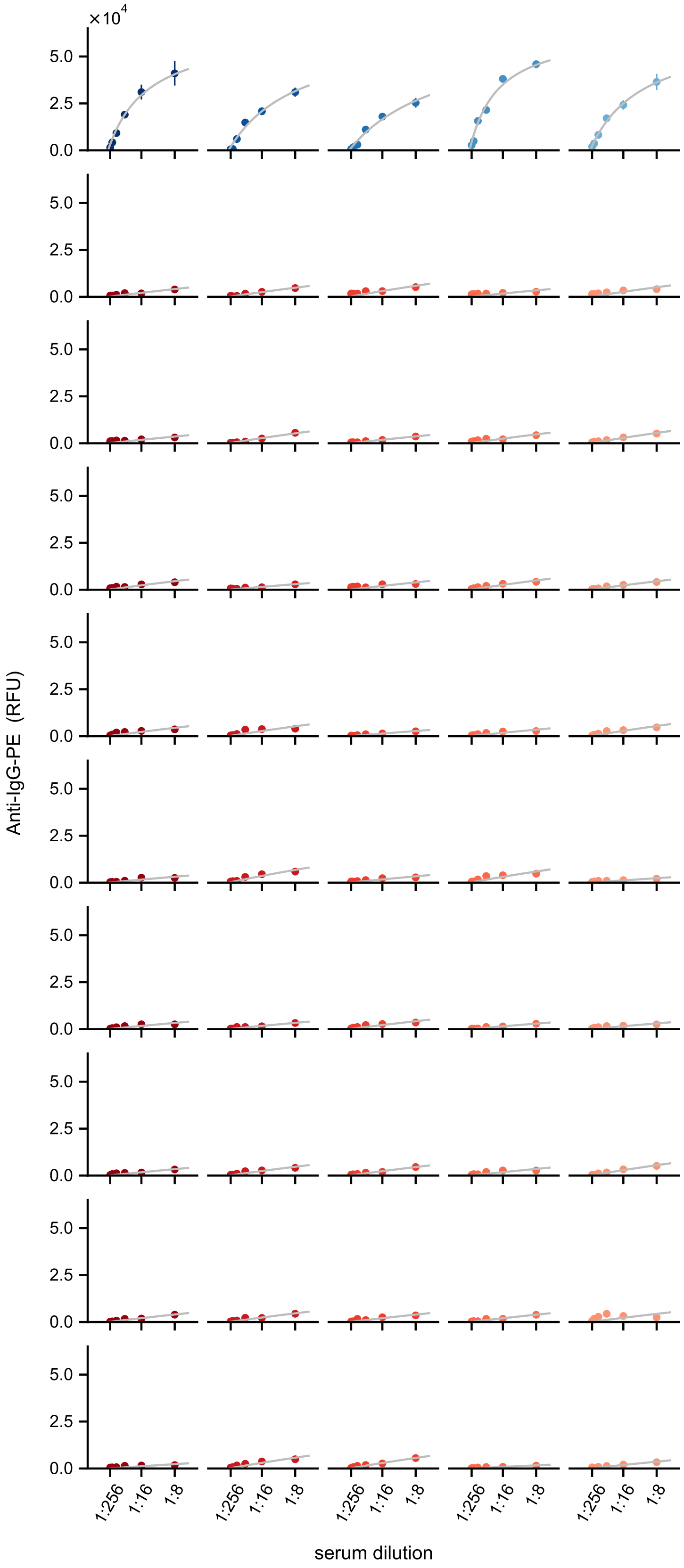

Fig. S6. Complete serum dilution data.

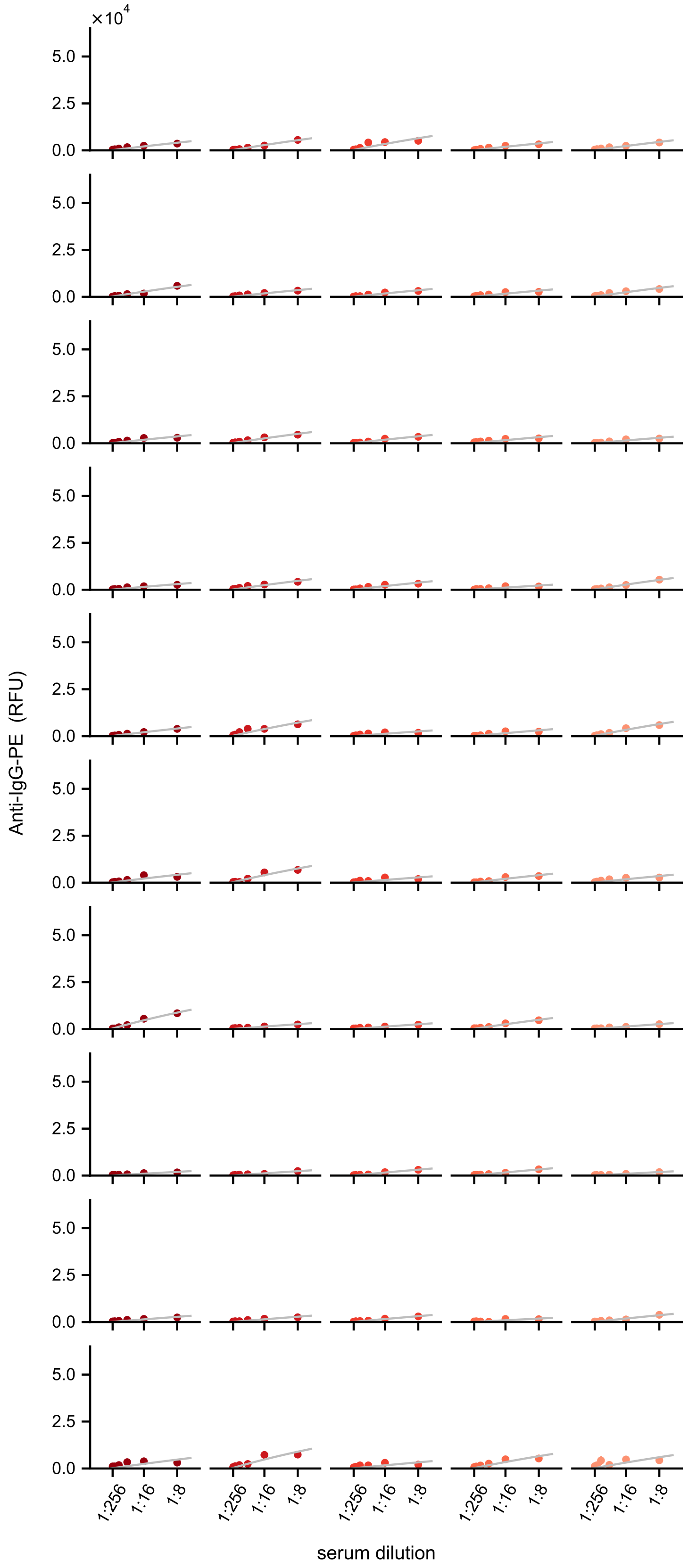

Fig. S6. Complete serum dilution data.

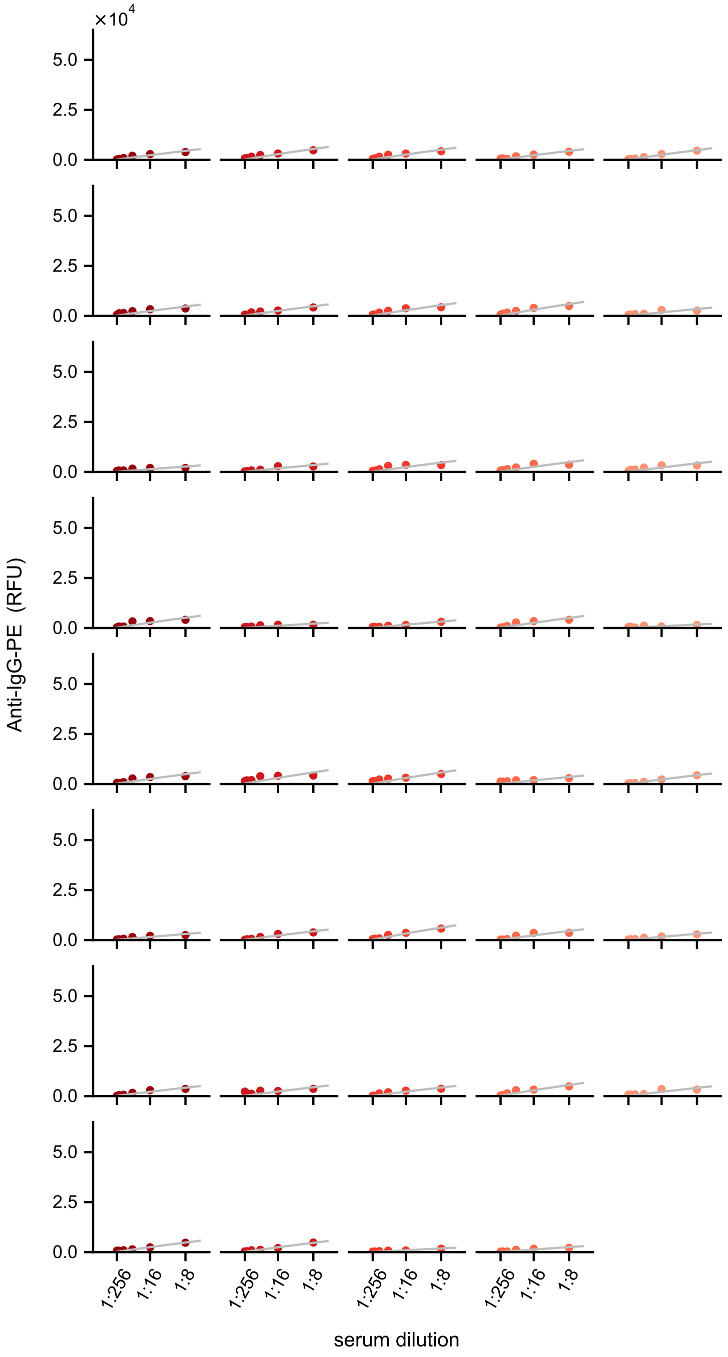

Fig. S6. Complete serum dilution data.

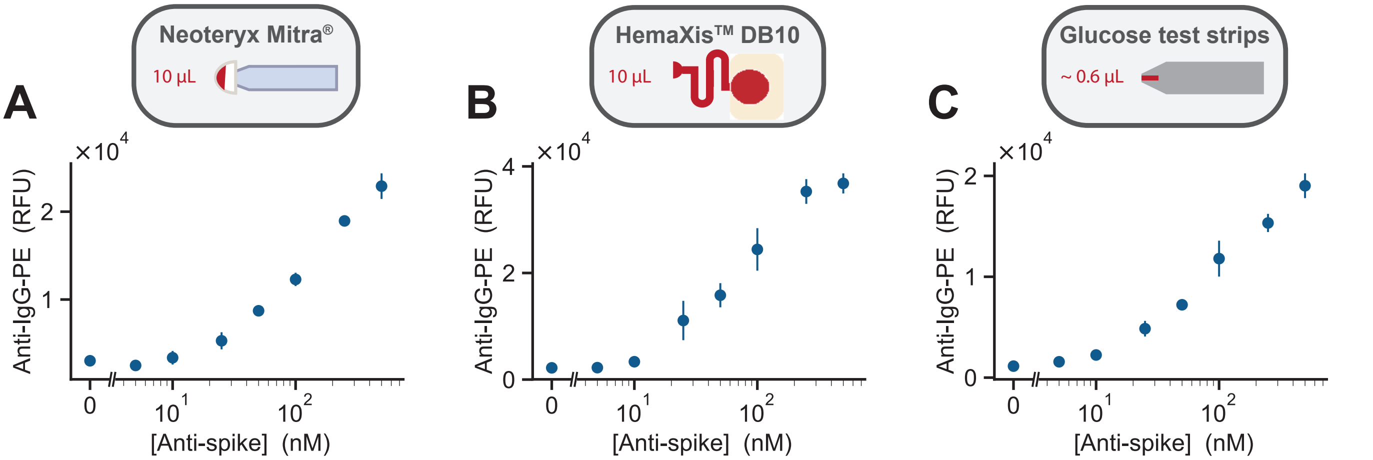

Fig. S7. Detection of anti-spike IgG in whole blood. (A-C) Non-normalized on-chip anti-IgG-PE signal versus the concentration of anti-spike-IgG in whole blood sampled using each of the three methods: Mitra®, HemaXis^{TM} DB10, and glucose test strips (shown in this order from left to right).

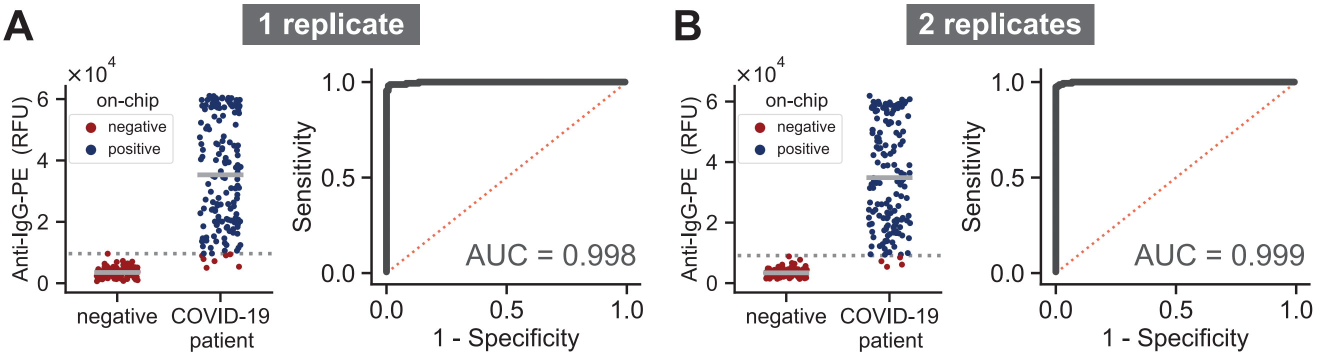

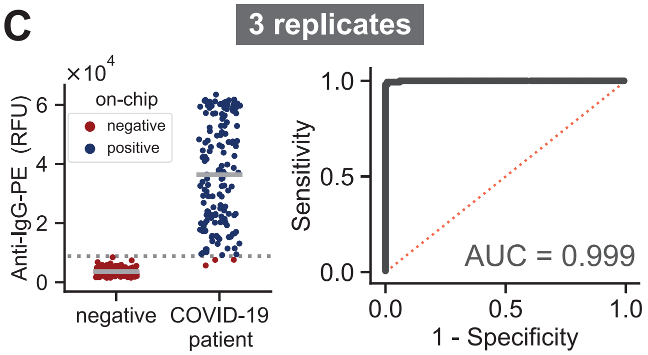

D

replicates	specificity	95% CI	sensitivity	95% CI
1	100%	97-100%	95%	90-97%
2	100%	97-100%	97%	93-98%
3	100%	97-100%	98%	94-99%

Fig. S8. Technical replicates for ultra-low volume whole blood sampling methods. (A-C) On-chip anti-IgG-PE signal versus the concentration of anti-spike-IgG in whole blood for three technical replicates sampled using each of the three methods: Mitra®, HemaXis™ DB10, and glucose test strips (shown in this order from left to right). **(D)** Coefficient of variation versus the concentration of anti-spike-IgG for each blood sampling method.

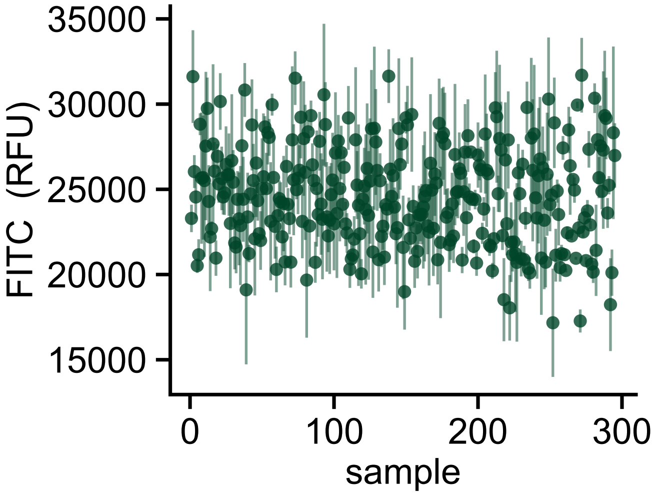

Fig. S9. FITC spotting tracer. FITC-dextran (10 kDa) signal for each serum sample (1:8 dilution). Images were acquired in the spotting chamber after the sample had been resolubilized.

SI Dataset S1 (patient_serum_data.xlsx)

SI Dataset S2 (patient_blood_data.xlsx)

References

1. D Wrapp, et al., Cryo-EM structure of the 2019-nCoV spike in the prefusion conformation. *Science* **367**, 1260–1263 (2020).
2. C Klumpp-Thomas, et al., Standardization of enzyme-linked immunosorbent assays for serosurveys of the SARS-CoV-2 pandemic using clinical and at-home blood sampling. *medRxiv* (2020).
3. S Rockel, M Geertz, SJ Maerkl, MITOMI: A Microfluidic Platform for In Vitro Characterization of Transcription Factor–DNA Interaction. *Methods Mol. Biol.* **786**, 97–114 (2011).
4. JA White, AM Streets, Controller for microfluidic large-scale integration. *HardwareX* **3**, 135–145 (2017).
5. K Brower, et al., An Open-Source, Programmable Pneumatic Setup for Operation and Automated Control of Single and Multi-Layer Microfluidic Devices. *HardwareX*, 1 – 37 (2017).
